# Systematic analysis of adverse reaction signals induced by leflunomide, upadacitinib, and baricitinib in the treatment of rheumatoid arthritis: a FAERS database analysis

**DOI:** 10.3389/fmed.2026.1753446

**Published:** 2026-07-21

**Authors:** Jizheng Dang, Li Shu

**Affiliations:** Department of Pharmacy, University-Town Hospital of Chongqing Medical University, Chongqing, China

**Keywords:** rheumatoid arthritis, leflunomide, upadacitinib, baricitinib, adverse drug events

## Abstract

**Purpose:**

Rheumatoid arthritis (RA) is a widely recognized chronic inflammatory disease. Leflunomide, upadacitinib, and baricitinib are frequently used to treat inflammatory diseases, including RA, although understanding of their associated adverse drug events (ADEs) remains limited. This study analyzed ADEs reported for these drugs based on the United States FAERS database.

**Methods:**

Data were extracted from the FAERS database, including ADE reports from January 2004 to December 2024. ADE reports related to leflunomide, upadacitinib, and baricitinib were described and categorized using the preferred term (PT) and system organ class (SOC) based on the International Dictionary of Medical Terms (MedDRA). The baseline characteristics of leflunomide, upadacitinib, and baricitinib were analyzed; the readxl R package was used for variable analysis. The ggplot2 R package was used to plot temporal trend line charts of ADE reports for each drug used for RA treatment.

**Results:**

The baseline analysis showed that the number of ADE reports was 10,445 for leflunomide, 27,433 for upadacitinib, and 2,638 for baricitinib. The number of ADE reports for baricitinib following RA treatment was highest in 2019, whereas the number of reports for leflunomide peaked in 2020, whereas those relative to upadacitinib reached their peak in 2022. Baricitinib-related ADE reports in RA treatment included infections and infestations and blood and lymphatic system disorders. Leflunomide-related ADE reports involved hepatic disorders and congenital, familial, and hereditary diseases. Upadacitinib-related ADE reports included surgical and medical procedures.

**Conclusion:**

This study, based on the FAERS database, identified ADEs in leflunomide, upadacitinib, and baricitinib, to provide a reference for improving drug safety and therapeutic efficacy.

## 1 Introduction

Rheumatoid arthritis (RA) constitutes a chronic systemic autoimmune disorder pathognomonically characterized by symmetric polyarticular inflammation, which culminates in structural deterioration, functional impairment, and systemic complications (1). As a systemic autoimmune pathology associated with chronic inflammatory cascades, RA exhibits multi-organ involvement that includes the cardiac, renal, pulmonary, gastrointestinal, ocular, dermal, and neurological systems (2). Moreover, the etiology of this idiopathic disease primarily implicates genetic determinants, whereas environmental factors such as tobacco exposure, particulate matter inhalation, and microbiota dysbiosis constitute significant pathogenic contributors (3). Contemporary therapeutic paradigms encompass pharmacotherapy, physiotherapy, and surgical interventions, although these approaches frequently entail adverse effects (AEs) and demonstrate substantial heterogeneity in interindividual efficacy (4). Thus, mining AE signals of antirheumatic drugs provides robust references for rational clinical pharmacotherapy and medication surveillance.

Methotrexate (MTX) remains the ‌first-line therapeutic cornerstone‌ among conventional synthetic disease-modifying antirheumatic drugs (csDMARDs) for the initial management of RA. In cases where MTX contraindications or intolerance warrant alternative regimens, leflunomide has emerged as a ‌viable therapeutic alternative (5)‌. Classified as a disease-modifying antirheumatic agent, leflunomide exerts its ‌immunomodulatory effects‌ through selective inhibition of dihydroorotate dehydrogenase (DHODH) a pivotal mitochondrial enzyme in the *de novo* pyrimidine biosynthesis pathway (6). This enzymatic suppression disrupts ‌lymphocytic proliferation kinetics‌ and attenuates hyperactivated immune responses by altering pyrimidine-dependent RNA/DNA synthesis in rapidly dividing T-lymphocytes. Following a suboptimal therapeutic response or intolerance to csDMARDs, targeted synthetic DMARD (tsDMARDs) can be incorporated into combination regimens. Within this therapeutic class, ‌upadacitinib‌ and ‌baricitinib‌ assume pivotal therapeutic roles in contemporary RA management (7). Upadacitinib‌, a selective Janus kinase (JAK) inhibitor, exerts its pharmacodynamic effects through targeted suppression of JAK enzymatic activity. This molecular intervention ‌inhibits phosphorylation of downstream effector proteins, consequently disrupting cytokine signal transduction within inflammatory pathways critically implicated in immune-mediated pathologies (8). Baricitinib, a potent JAK inhibitor, exerts its therapeutic effects through the selective antagonism of JAK1 and JAK2 isoforms. This targeted inhibition disrupts phosphorylation-dependent signal transduction cascades within the JAK/STAT signaling pathway, thereby attenuating downstream signaling of pivotal proinflammatory cytokines including interleukin (IL)-6, interferon-gamma (IFN-*γ*), and IL-23 (9).

Although all three drugs are effective treatment options for RA, leflunomide is a csDMARD whereas baricitinib (JAK1/2 inhibitor) and upadacitinib (highly selective JAK1) are tsDMARDs with different mechanisms of action, which may lead to distinct AEs. Thus, analyzing the safety signals for each drug separately can help clarify the real-world safety features of traditional drugs and newer therapies. Moreover, the differing target selectivity of baricitinib and upadacitinib, although both JAK inhibitors, may also be useful for better understanding the safety heterogeneity among mechanistically similar agents.

The Food and Drug Administration (FDA) Adverse Event Reporting System (FAERS), ‌spearheaded by the U.S. FDA‌, constitutes a ‌globally recognized pharmacovigilance repository‌. This system perpetually records, systematizes, and scrutinizes‌ spontaneously submitted reports of adverse drug events (ADEs) originating from ‌domestic and international patients, healthcare practitioners, and manufacturing entities‌, thereby furnishing an ‌indispensable data foundation‌ for postmarketing pharmaceutical safety evaluations (10, 11). The sex-based analysis of AEs associated with upadacitinib reported in the FAERS database, highlights the importance of considering differences in AEs based on sex (12). Using the same database a previous study defined the varying reported safety signal profiles of leflunomide, upadacitinib, and baricitinib, highlighting the need for personalized evaluation of pharmacovigilance signals and careful patient selection in clinical practice (13). AE profiles‌ associated with leflunomide, upadacitinib, and baricitinib during RA management remain inadequately characterized, particularly in terms of long-term safety outcomes. Given the prolonged therapeutic trajectories‌ inherent in RA, treatment-emergent AEs such as hepatotoxicity, opportunistic infections, and hematological abnormalities can compromise therapeutic efficacy and patient quality of life. Consequently, ‌enhanced pharmacovigilance frameworks‌ and early identification of ADRs are of critical importance in clinical deployment.

This study conducted a comprehensive pharmacoepidemiologic investigation of ADRs associated with leflunomide, upadacitinib, and baricitinib, in the management of RA using FAERS data. The analysis used a rigorous retrospective pharmacovigilance approach, encompassing data from the first quarter of 2004 to the fourth quarter of 2024, with a 20-year study span that more fully reflected long-term drug safety trends. By integrating algorithms such as the reporting odds ratio.

(ROR), proportional reporting ratio (PRR), Bayesian confidence propagation neural network (BCPNN), and multi-item gamma poisson shrinker (MGPS) to analyze demographic and geographic distributions, the study revealed reporting profiles for leflunomide, upadacitinib, and baricitinib across different populations, regions, and time periods. This study provides a valuable reference for drug safety and treatment strategies for patients with RA in various countries and regions.

## 2 Materials and methods

### 2.1 Data sources

The raw data for this study were sourced from the FAERS database,^1^ which is updated quarterly and is freely available to the public free in American Standard Code for Information Interchange (ASCII) or Extensible Markup Language (XML) format (14). ADE reports from the first quarter of 2004 to the fourth quarter of 2024 were selected. Three drugs (leflunomide, upadacitinib, and baricitinib) were used as search terms to extract ADE reports drugs used for RA treatment. According to FDA recommendations, downloaded ASCII data packets were processed and imported into the R software (v4.2.2) for data cleaning. Data were classified by preferred term (PT) and system organ classes (SOC) to facilitate analysis. In the FAERS database, AE reports were retrieved with the drug role of “primary suspect” to ensure that only reports directly related to the target drug were included. Furthermore, ADE reports related to leflunomide, upadacitinib, and baricitinib were described and classified using PT and SOC in MedDRA, making the data more standardized and systematic for subsequent analysis.

**Figure 1:**
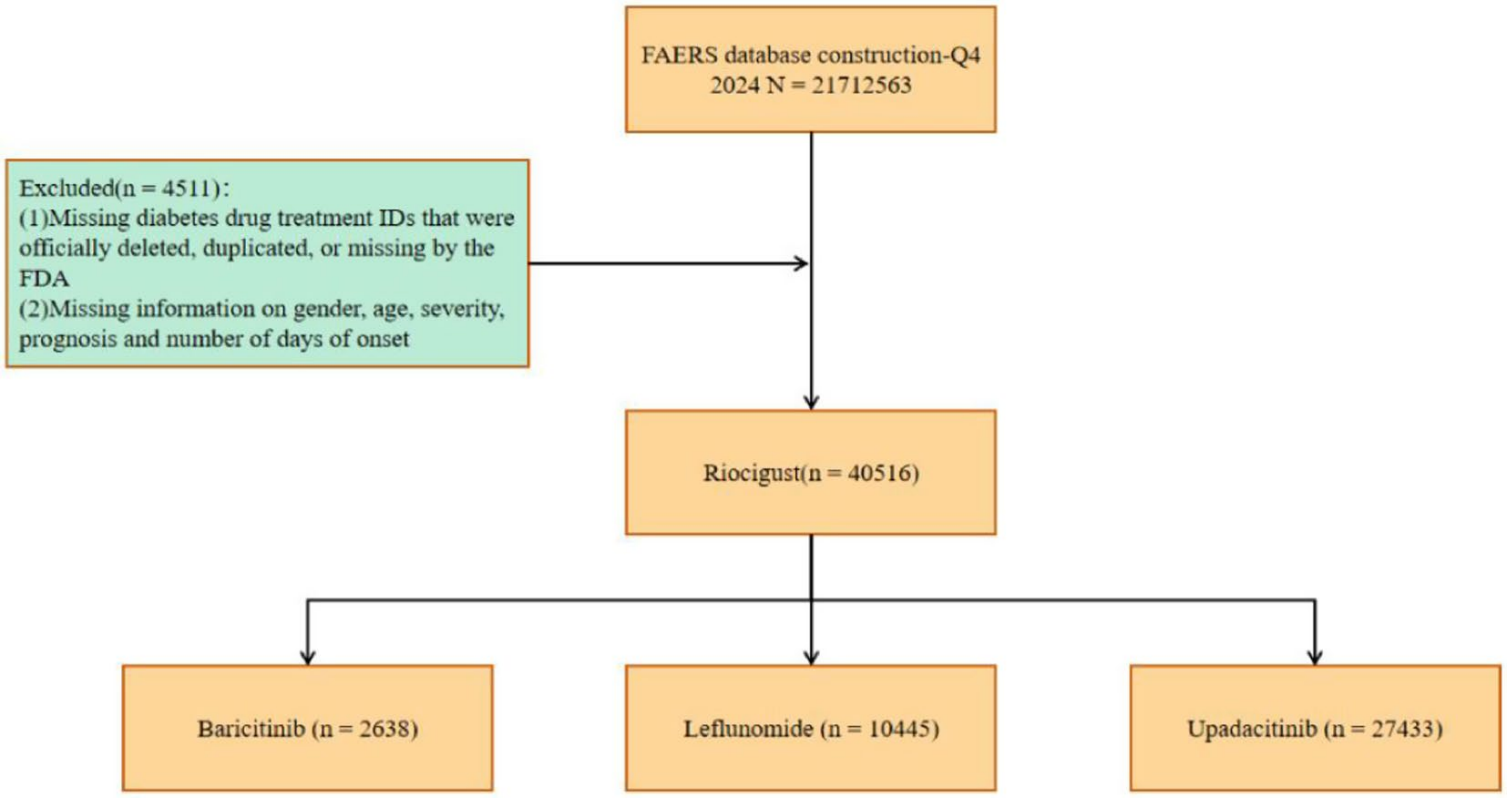
Flow diagram for inclusion and exclusion of the FAERS reports.

The exclusion criteria of this study were as follows (15): (1) AE IDs of drug treatments for leflunomide, upadacitinib, and baricitinib that had been officially deleted, duplicated, or missing by the FDA and (2) reports with incomplete or inaccurate information on sex, age, reporter country, outcome, or drug indication. After applying these criteria, 40,516 ADE reports were retained for analysis: 10,445 for leflunomide, 27,433 for upadacitinib, and 2,638 for baricitinib. The detailed research procedure is illustrated in Figure 1.

### 2.2 Data extraction using disproportionality analysis

Multiple sophisticated algorithms were adopted for disproportionality analysis to thoroughly explore and analyze the relationships between drugs and potential ADRs. The disproportionality method is a pivotal approach, with the ROR and PRR serving as its two primary metrics. The fundamental principle of the disproportionality method was to use a four grid table (comprising values a, b, c, and d representing different counts of drug-related and event-related reports as defined in the study) to compare the occurrence frequencies of target drugs and target events with their respective background frequencies (Table 1). To enhance the reliability of the results and minimize the interference of false-positive signals, ROR and PRR were used in combination in this study. Furthermore, the BCPNN method and the MGPS algorithms were also employed to detect ADE signals. Their calculation formulas and threshold criteria are shown in Supplementary Table S1. The criteria for determining effective ADE signals required that all of the following thresholds were satisfied simultaneously: the number of ADE reports ≥ 3; the lower limit of the 95% confidence interval (CI) of the ROR value> 1; PRR ≥ 2 and *χ*^2^ ≥ 4; EBGM lower 95% confidence bound (EBGM_05_) > 2; and information component (IC_025_) > 0.

**Table 1:** Fundamental principle of the disproportionality method.

Drug	Number of target event reports	Number of other event reports	Total
Target drug	a	b	a + b
Other drugs	c	d	c + d
Total	a + c	b + d	a + b + c + d

### 2.3 Statistical analysis

To fully explore the clinical application of leflunomide, upadacitinib, and baricitinib in the treatment of RA, we screened relevant data from the FAERS database (2004–2024) to obtain basic information on these drugs and to identify ADEs that occurred during their use in the treatment of RA. The “readxl” R package (v1.4.2)^2^ was used to read the data, and baseline statistical analyses of the ADE reports of the three drugs were performed. The “ggplot2” R package (v3.4.4) (16) was employed to plot line charts depicting the temporal trends of ADE reports for each drug in the treatment of RA. Then, SOC and PT classification of the involved ADEs, using the ROR, PRR, BCPNN, and MGPS methods were adopted for analysis.

In addition, to explore the potential factors associated with reporting for ADEs associated with baricitinib, leflunomide, upadacitinib in the treatment of RA and analyze the reporting patterns of specific subgroups of patients, a stratified subgroup analysis was conducted in patients based on the FAERS data from the first quarter of 2004 to the fourth quarter of 2024. The patients were stratified by sex (male and female), age (0–64 years, 65–120 years), type of reporter (consumer and healthcare professional) and severity of event (serious and non-serious) to assess the association of these factors with ADE reporting. The ROR was used to determine the strength of the association of the ADE signal detection. The higher the ROR value, the stronger the reporting association.

All analyses were implemented with R (v4.2.2).

## 3 Results

### 3.1 Baseline statistics and temporal trend change analysis for ADEs associated with leflunomide, upadacitinib and baricitinib for the treatment of RA

This data distribution and analysis results of ADEs associated with leflunomide, upadacitinib, and baricitinib for the treatment of RA are summarized in Supplementary Table S2. In terms of sex, the proportion of ADEs reports in females (F) was higher than in males (M) for leflunomide (75.0%), upadacitinib (78.0%), and baricitinib (76.2%). In terms of body weight, patients with a weight range of 50–100 kg accounted for a higher proportion of ADEs. In terms of age, the 65–85 year-old group reported more ADEs reports of baricitinib (39.2%), whereas those aged 18–64.9 years comprised a higher proportion for leflunomide (43.1%) and upadacitinib (29.8%) ADEs. In terms of reporter type, consumers (CN) accounted for a higher proportion of AE reports related to baricitinib (51.1%) and upadacitinib (72.9%), whereas physicians (MD) accounted for a higher proportion of reports relative to leflunomide (33.7%). In terms of reporter country, the U.S. accounted for 37.5% of baricitinib reports and 68.4% of upadacitinib reports, whereas Canada accounted for 51.7% of leflunomide reports. The FAERS data regarding baricitinib, leflunomide and upadacitinib applied to the treatment of RA since the database was established.

### 3.2 Analysis of the temporal changes for ADEs associated with leflunomide, upadacitinib, and baricitinib for the treatment of RA, as well as analyses based on the levels of SOC and PT

Statistical analysis of ADE reports for the treatment of RA with baricitinib, leflunomide, and upadacitinib registered by the FAERS database showed that from 2004 to 2017, only ADE reports related to leflunomide treatment for RA were available. After 2017, reports of ADEs associated with baricitinib and upadacitinib for RA began to markedly increase. In 2019, the number of reports of ADEs arising from baricitinib treatment for RA was the highest, followed by a slow decline. In 2020, leflunomide had the highest number of RA-related ADE reports, after which the quantity decreased. In 2022, upadacitinib reached the highest number of reports of ADE among all drugs for RA, with a subsequent decline in reports by 2024 (Supplementary Figure 1).

Among the ADRs reports classified by SOC, 4 valid ADE signals related to baricitinib in the treatment of RA were detected among the 27 levels of SOC: Infections and infestations, Blood and lymphatic system disorders: Vascular disorders; Neoplasms benign, malignant and unspecified (Supplementary Table S3). Two valid ADE signals related to leflunomide in the treatment of RA were detected: Hepatobiliary disorders and congenital, familial and genetic disorders (Supplementary Table S4). A valid ADE signal related to upadacitinib was detected associated with the treatment of RA: Surgical and Medical Procedures. Taking “Surgical and Medical Procedures” as an example, upadacitinib had 7,022 reports (ROR 4.83, 95% CI 4.71–4.95, PRR 4.5; *χ*^2^ 17,538.36; EBGM_05_ 4.06; and IC_025_ 2.01) (Supplementary Table S5). At the PT level, a total of 205 valid ADE signals for baricitinib were associated with the treatment of RA and were detected among the 1,454 PT levels. Considering “Therapy interrupted” as an example, baricitinib had 93 reports (ROR 5.83, 95% CI 4.75–7.16; PRR 5.75; and *χ*^2^ 362.59) (Supplementary Table S6). A total of 605 valid ADE signals for leflunomide in the treatment of RA were detected among 3,069 PT levels. Taking “Drug intolerance” as an example, leflunomide had 1,800 reports (ROR 3.97, 95% CI 3.78–4.17; PRR 3.92; *χ*^2^ 3,544.83) (Supplementary Table S7). A total of 628 valid ADE signals for upadacitinib in the treatment of RA were detected among the 4,231 PT levels. Considering “COVID-19” upadacitinib had 2,037 reports (ROR 6.9, 95% CI 6.58–7.24; PRR 6.76; *χ*^2^ 8,567.67) (Supplementary Table S8). All these values met the criteria for identifying valid ADE signals set in this study (i.e., number of ADE reports ≥ 3, ROR > 1, PRR ≥ 2, *χ*^2^ ≥ 4, EBGM_05_ > 2, IC_025_ > 0). Notably, these signal counts reflected differences in observation periods and reporting volumes; direct comparison across drugs was not appropriate.

### 3.3 Stratified disproportionality analysis by age, sex, reporter type, and event severity

**Figure 2:**
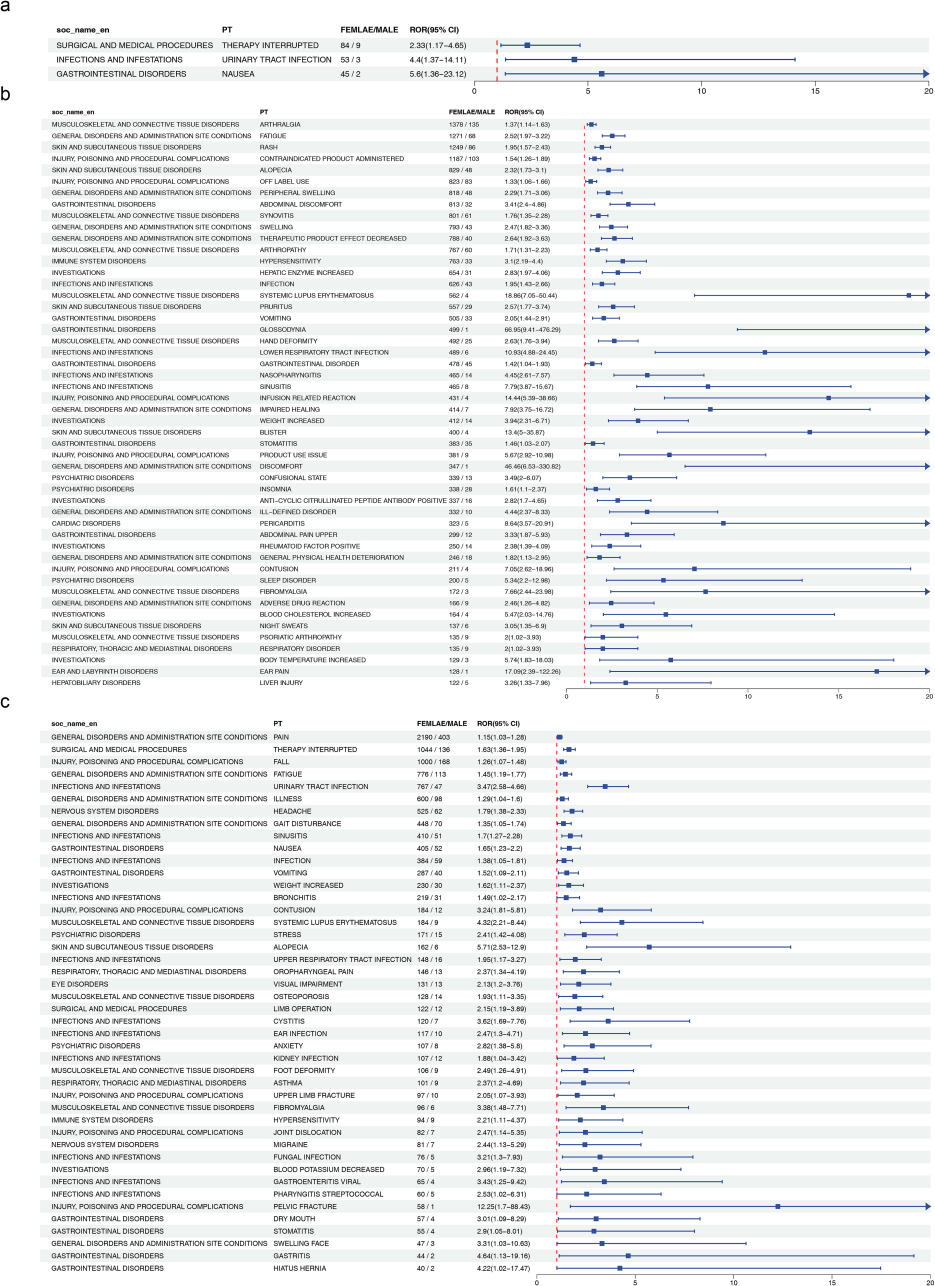
Different gender subgroups. From top to bottom: baricitinib **(a)**, leflunomide **(b)**, upadacitinib **(c)**. When the 95% CI of ROR for PT events did not include 1, it was considered that there were differences between different subgroups.

In the analysis of the sex subgroups of RA treatment, each of the three drugs exhibited a specific pattern of disproportionality signals at the PT level. Baricitinib showed disproportionality signals for therapy interrupted (ROR 2.33, 95% CI 1.17–4.65), urinary tract infection (ROR 4.40, 95% CI 1.37–14.11), and nausea (ROR 5.60, 95% CI 1.36–23.12) (Figure 2a). Leflunomide showed disproportionality signals for a wide range of PTs, encompassing various symptoms such as arthralgia (ROR 1.37, 95% CI 1.14–1.63) and fatigue (ROR 2.52, 95% CI 1.97–3.22), as well as rash (ROR 1.95, 95% CI 1.57–2.43), in addition to medication-related issues (Figure 2b). Upadacitinib showed disproportionality signals for pain (ROR 1.15, 95% CI 1.03–1.28), therapy interrupted (ROR 1.63, 95% CI 1.36–1.95), fatigue (ROR 1.45, 95% CI 1.19–1.77), and a variety of other symptoms and diseases (Figure 2c).

**Figure 3:**
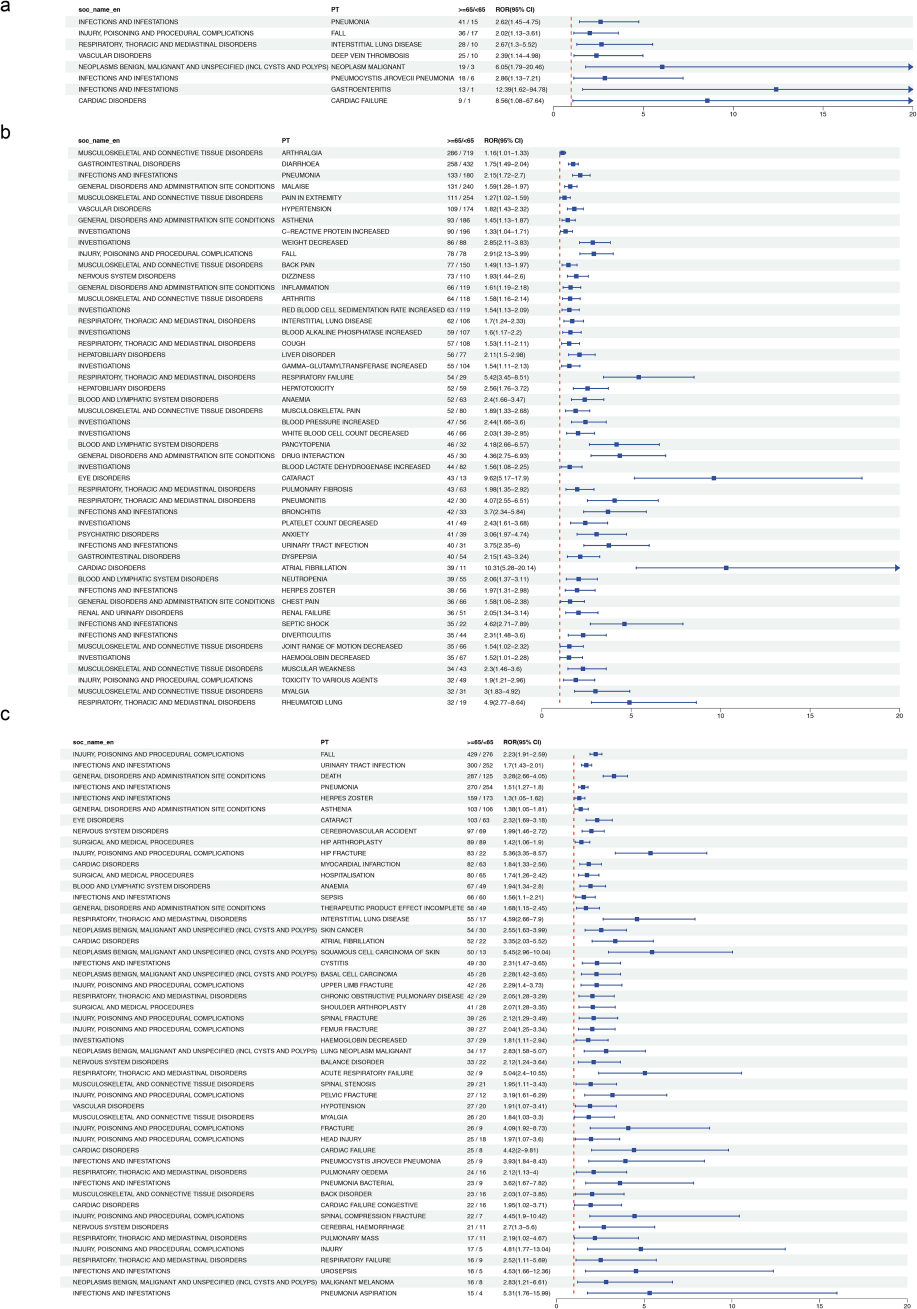
Different age subgroups. From top to bottom: baricitinib **(a)**, leflunomide **(b)**, upadacitinib **(c)**. When the 95% CI of ROR for PT events did not include 1, it was considered that there were differences between different subgroups.

In the age subgroup analysis of RA treatment, the 3 drugs showed different signals with highly significant disproportionality at the PT level. Baricitinib showed disproportionality signals for pneumonia (ROR 2.62, 95% CI 1.45–4.75), malignant neoplasm malignant (ROR 6.05, 95% CI 1.79–20.46), and other conditions (Figure 3a). Leflunomide showed disproportionality signals for a wide range of symptoms and indicators, including not only common symptoms such as arthralgia (ROR 1.16, 95% CI 1.01–1.33), but also abnormalities of important physiological indicators (e.g., hypertension [ROR 1.82, 95% CI 1.43–2.32]) and organ damage, as well as signals for herpes zoster (ROR 1.97, 95% CI 1.31–2.98) (Figure 3b). Upadacitinib showed disproportionality signals for SAEs. Apart from urinary tract infection (ROR 1.70, 95% CI 1.43–2.01), it also included deaths (ROR 3.28, 95% CI 2.66–4.05) and myocardial infarction (ROR 1.84, 95% CI 1.33–2.56), along with reports associated with treatment-related outcomes such as hospitalization (ROR 1.74, 95% CI 1.26–2.42) (Figure 3c).

**Figure 4:**
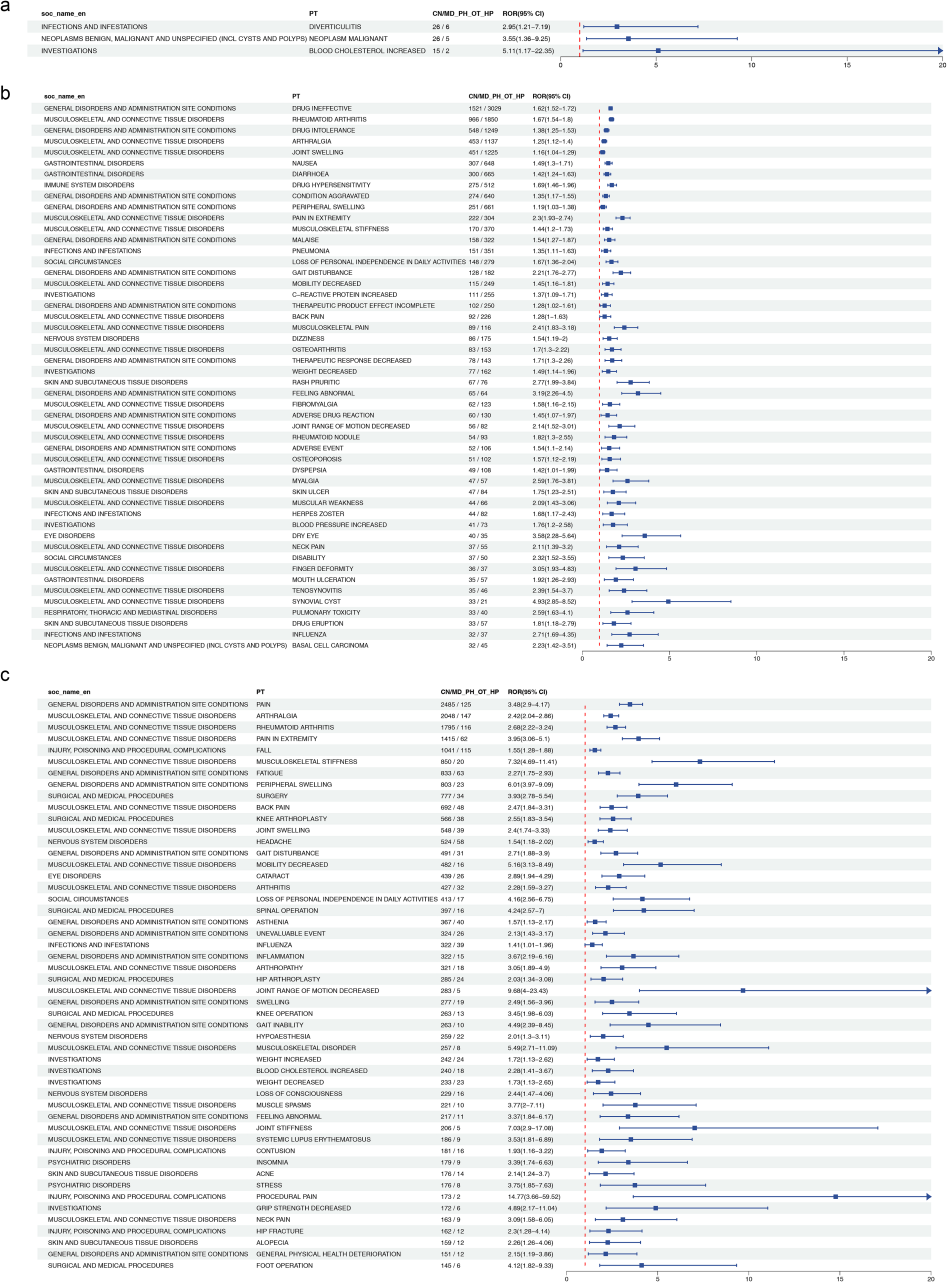
Different reporter type subgroups. From top to bottom: baricitinib **(a)**, leflunomide **(b)**, upadacitinib **(c)**. When the 95% CI of ROR for PT events did not include 1, it was considered that there were differences between different subgroups.

In the analysis of the reporter type subgroup of RA treatment, all three drugs exhibited distinct characteristics in terms of high significant disproportionality signals at the PT level. Baricitinib showed few disproportionality signals, with signals only observed in diverticulitis (ROR 2.95, 95% CI 1.21–7.19) and higher blood cholesterol levels (ROR 5.11, 95% CI 1.17–22.35) (Figure 4a). Leflunomide had a broad range of disproportionality signals, including those not only related to medication issues such as ineffective drug outcomes (ROR 1.62, 95% CI 1.52–1.72) and common symptoms like arthralgia (ROR 1.25, 95% CI 1.12–1.40), but also signals for specific AEs (e.g., pulmonary toxicity [ROR 2.59, 95% CI 1.63–4.10]) (Figure 4b). Upadacitinib showed disproportionality signals mainly focusing on musculoskeletal system symptoms (e.g., pain in extremities [ROR 3.95, 95% CI 3.06–5.10]), along with reporting associations with treatment-related outcomes like surgery (ROR 3.93, 95% CI 2.78–5.54) and signals for systemic lupus erythematosus (ROR 3.53, 95% CI 1.81–6.89) (Figure 4c). Additionally, upadacitinib and leflunomide both showed disproportionality signals for influenza.

**Figure 5:**
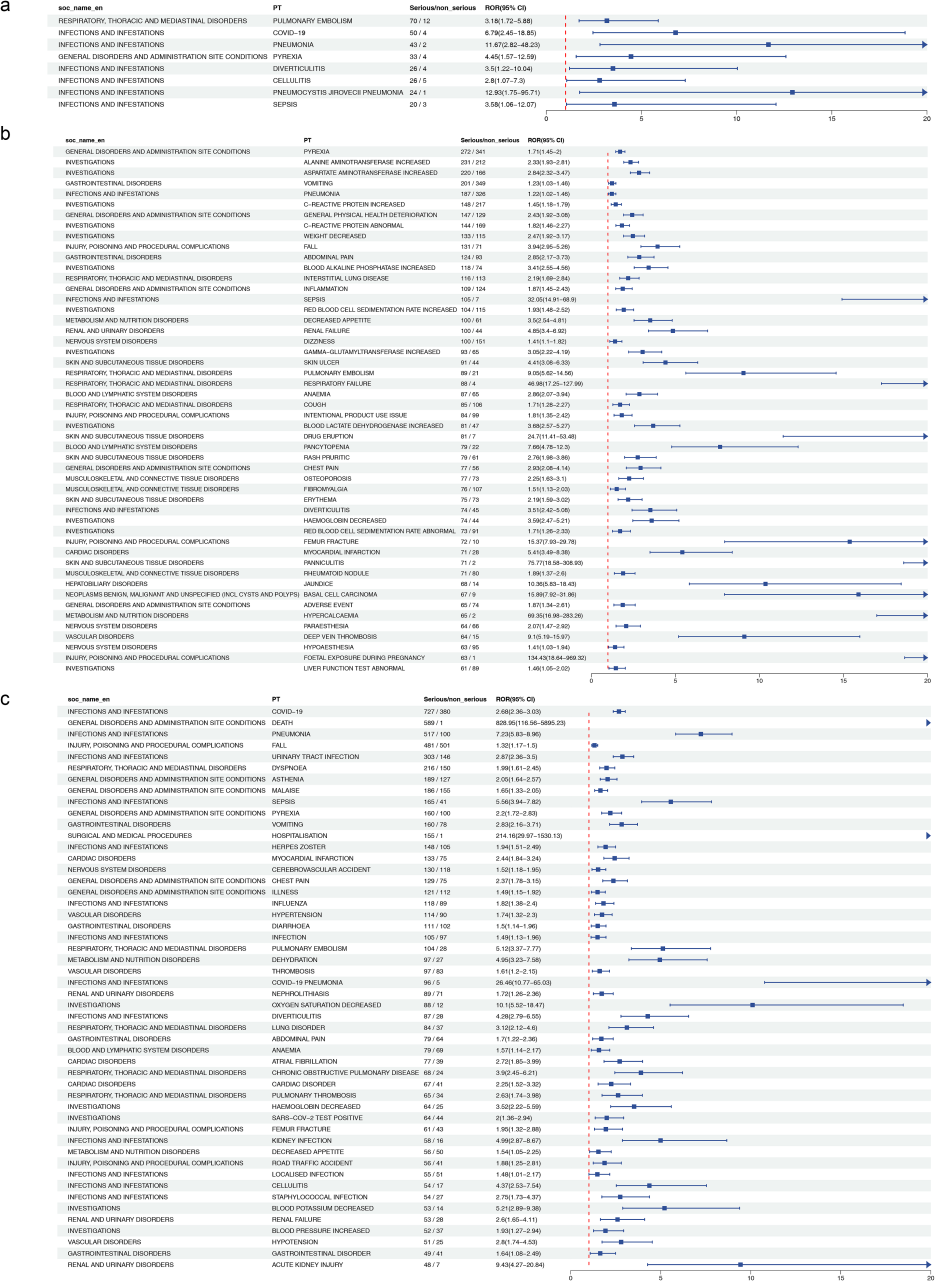
Different event severity stratification subgroups. From top to bottom: baricitinib **(a)**, leflunomide **(b)**, upadacitinib **(c)**. When the 95% CI of ROR for PT events did not include 1, it was considered that there were differences between different subgroups.

In the stratification of event severity for RA treatment, baricitinib showed disproportionality signals at the PT level only for a few specific AEs, such as pulmonary embolism (ROR 3.18, 95% CI 1.72–5.88) and sepsis (ROR 3.58, 95% CI 1.06–12.07) (Figure 5a). Leflunomide showed the broadest range and largest number of disproportionality signals across AEs, including pneumonia (ROR 1.22, 95% CI 1.02–1.46) (Figure 5b). Upadacitinib showed disproportionality signals at the PT level for AEs like COVID-19 (ROR 2.68, 95% CI 2.36–3.03) and its associated pneumonia (ROR 7.23, 95% CI 5.83–8.96) (Figure 5c).

In the subgroups of sex, age, and reporter type as well as in the stratification of the severity of the event of the RA treatment, baricitinib, leflunomide, and upadacitinib showed different patterns of disproportional signals at the PT level. Specifically, baricitinib showed signal events for a limited number of PTs; leflunomide showed signal events for a wide range of PTs; and upadacitinib showed signal events for specific symptoms and severe outcomes. In addition, overlapping signals for events such as pneumonia and interruption of therapy were observed across the three drugs.

## 4 Discussion

Leflunomide, upadacitinib, and baricitinib constitute first-line therapeutic agents for the management of RA. ADRs reported in association with these pharmacotherapies can compromise therapeutic outcomes and cause serious complications in affected patients. The FAERS aggregates global spontaneous reports of ADEs, establishing an evidence-based foundation for optimizing clinical pharmacotherapy regimens through multidimensional signal detection (17). This study, using multidimensional evaluation through the FAERS system, assessed the ADEs of leflunomide, upadacitinib, and baricitinib in RA treatment, incorporating demographic profiling, geographic distribution analysis, and the dissection of ADE categories at the SOC and PT levels. The analysis revealed the respective reporting patterns of the three drugs in the FAERS database, providing information that may be useful for future drug safety surveillance and regulatory frameworks.

Regarding sex-based differences, this study showed that female patients had a higher number of ADR reports compared with their male counterparts. This pattern was consistent with the fact that women had a much higher incidence of RA than men. Data from the 2021 Global Burden of Disease Study showed that among working-age adults between 15 and 49 years of age, the age-standardized incidence of RA was 228.3/100,000 in women and 81.0/100,000 in men. In this age group, women were~2.8 times more likely than men to be diagnosed with RA, and women accounted for 73% of all RA cases (18–20). This finding underscores that clinicians should develop more tailored treatment plans and enhance monitoring for patients undergoing RA therapy. In terms of age stratification, patients aged 65–85 years accounted for a disproportionately higher share of baricitinib-associated ADE reports. This phenomenon may relate to the decline in immune function among the older patients (21). It is also influenced by comorbidities, biological vulnerability, comorbidity burden, polypharmacy, ‌prescribing pattern preferences (22), and age-related differences in reporting behavior (23). These factors may act independently or jointly to affect the distribution of this signal in the older population. Distinguishing the independent contribution of each factor is difficult using FAERS data alone. In contrast, leflunomide and upadacitinib demonstrated disproportionate representation of ADE reports among patients aged 18–64.9 years (24) a phenomenon potentially influenced by a multi-factorial interplay among reproductive toxicity profiles, frontline therapeutic algorithms, and immune response dynamics. This suggests that clinicians can tailor treatment strategies according to patient age to balance efficacy and safety. Age subgroup analysis used only reports with complete age records. Nonetheless these results require confirmation with more complete demographic data and should be validated with real data to better characterize age-related AE signals.

Pharmacovigilance data show that adverse drug event (ADE) reports for leflunomide in rheumatoid arthritis (RA) were recorded only before 2017. Thereafter, reports for baricitinib and upadacitinib increased progressively, with reports for the three drugs peaking in 2019, 2020, and 2022, respectively. The peak in baricitinib reports may be related to an expanded indication population after its approval. The rising number of older RA patients may have increased drug exposure, but this speculation requires confirmation from actual prescription data (25) leflunomide, approved in 1998 as a first-line disease-modifying antirheumatic drug, has garnered a substantial accumulation of reports, reflecting the expected outcome of long-term clinical use. As clinicians gained experience, pharmacovigilance detectability improved (26). The surge in upadacitinib reports relates to its 2022 approval for early RA and to increased use in combination with biologics. Complex, multi-drug treatment scenarios may have promoted reporting of heterogeneous ADEs. After 2022, ADE reports for all three drugs declined markedly, which may be related to changes in prescribing patterns following the FDA and Enterprise management associates (EMA) black-box warnings for JAK inhibitors issued in 2021–2022 (27). Furthermore‌‌, as a voluntary reporting system, FAERS may see reduced reporting of common or minor events after safety signals are identified, and some patients may switch treatments, which reduces exposure to the initial drug (28). This interpretation of the temporal trend is only preliminary speculation based on report counts and lacks support from direct prescription or medication data. Future studies should incorporate drug-use data for further validation.

At the SOC level, the categories of Infections and infestations, as well as Blood and lymphatic system disorders, were the most prevalent for baricitinib-associated ADEs in the management of RA. Persistent elevation of proinflammatory cytokines, particularly TNF-*α*, IL-6, and IL-1—in patients with RA primarily drives immune cell dysregulation, which includes diminished neutrophil chemotaxis and aberrant T-cell/B-cell activation, culminating in compromised antimicrobial defenses (29). In addition, RA itself and its therapeutic agents can alter hematological components and lymphatic systems through various mechanisms. Common manifestations include anemia, leukopenia, thrombocytic anomalies, and elevated lymphoma risk (30). Hence, these manifestations can be attributed to the ‌dual effects‌ of inherent immune dysregulation in RA and pharmacological interventions, which collectively contribute to the observed reporting of infections and hematological ADEs during RA therapy. This study also found a strong disproportionality signal linking upadacitinib to COVID-19 (2,037 cases reported ROR 6.9). Although upadacitinib, a selective JAK1 inhibitor, can theoretically affect antiviral signaling and some studies have suggested that JAK inhibitors weaken the host antiviral immune response (31, 32), interpreting this signal requires consideration of multiple factors. First, the study’s data collection period overlapped with that of the global COVID-19 pandemic, and clinicians and the public increased testing and surveillance for COVID-19. Under those conditions, the number of reported COVID-19–related events in spontaneous reporting systems is amplified, which may have worsened reporting bias (33). Second, patients with RA have a baseline infection risk that is higher than that of the general population because of immune dysregulation and frequent concomitant use of immunosuppressants. Therefore, the validity of this association requires confirmation in well-designed pharmacoepidemiologic studies that fully adjust for confounding factors.

In addition, this study observed imbalance signals of infections and blood and lymphatic system diseases at the system organ classification level with regard to baricitinib; the reporting rate was higher among patients aged ≥65 years. This finding is broadly consistent with existing regulatory safety concerns regarding JAK inhibitors, including risks of severe infections, hematological abnormalities, and cardiovascular events. The FDA’s prescribing information for baricitinib highlights serious infections, and the EMA recommends monitoring older patients; our observed patterns are compatible with these communications. Regarding leflunomide, this study found that drug intolerance was the most common AE, which was reported relatively frequently among young and middle-aged adults. These findings are broadly consistent with the EMA’s long-standing hepatotoxicity concerns. In addition to being compatible with existing regulatory concerns, the subgroup analysis of this study can provide a reference for optimizing dosage guidelines for young and middle-aged adults. Regarding upadacitinib, this study detected a safety signal indicating an increased incidence of serious cardiovascular AEs, including myocardial infarction, and observed a marked rise in AEs reports following the 2022 indication expansion. This finding is compatible with the FDA black-box warning on cardiovascular risk and the EMA conditional marketing authorization. Through subgroup and severity stratification analyses, this study identified imbalance signals for baricitinib and upadacitinib that are compatible with the safety concerns highlighted in FDA and EMA communications. Data stratified by age, indication, and other key dimensions can provide clinicians with reference points for decision-making in specific patient populations. In contrast, analysis of leflunomide showed that reports of hepatotoxicity were more widely distributed across patient subgroups. It is important to note that these findings from FAERS disproportionality analyses are signal detection results and should not be interpreted as independent validation of regulatory warnings; rather, they reflect reporting patterns that are consistent with previously recognized safety concerns.

The study indicates that baricitinib accounts for a large share of AE reports in patients aged 65–85 years, with infection and disorders of the blood and lymphatic system emerging as the most prominent signals at the system-level. Clinicians should consider baseline immune status and infection-related complications in older patients, monitor complete blood count and infection symptoms (fever, cough) during therapy, and evaluate concomitant use of other immunosuppressive agents. Furthermore, the study showed that leflunomide was associated with a wide range of AEs and a higher rate of discontinuation due to intolerance, especially in patients aged 18–64 years. Clinicians should also assess baseline liver function and other comorbid conditions before starting therapy. The recommendation is to start low and gradually increase with doses as tolerated, monitor liver enzymes, watch for skin rash and gastrointestinal discomfort when using treatment, and adjust the regimen as needed. These findings of this study derived from the signal analysis of the FAERS database and therefore indicate potential safety signals rather than causality. The true risks and benefits require confirmation in prospective studies or using real-life data.

In this study, 20 years of FAERS data were surveyed from 2004 to 2024 to analyze AEs to three antirheumatic drugs (leflunomide, upadacitinib and baricitinib) and to analyze postmarketing safety signals for upadacitinib after a 2022 expansion of indications. Unlike prior studies that focused on a single JAK inhibitor or provided only short-term analyses (34), the long-term data coverage of this study, revealed temporal trends in AE reports for three drugs and their potential associations with regulatory actions. Methodologically, the study integrated four signal-detection algorithms—ROR, PRR, BCPNN, and MGP and included leflunomide, which has a different mechanism of action, as a control. Compared with earlier FAERS analyses that typically applied one or two algorithms (35), this study design strengthened the robustness of ADE signal identification. In addition, this study further revealed risk differences among subgroups by conducting multidimensional stratified signal analyses by age, sex, reporter type, and AE severity. For example, AE reports for baricitinib were concentrated in people older than 65 years, and infection signals were predominant. ADE reports for leflunomide were concentrated in the 18–64.9 year age group, and hepatotoxicity signals were predominant. Myocardial infarction and death signals appeared in the severe AE subgroup for upadacitinib. In summary, this study systematically presented the differential safety signal characteristics of three drugs across populations, time periods, and severity levels by using extended time coverage, multi-algorithm integration, and multidimensional stratified analysis, and it provided new evidence for individualized clinical drug selection and regulatory decision-making.

The present study had several limitations that should be acknowledged. First, FAERS is a spontaneous reporting system with biases such as under-reporting, duplicate reports, and reporter bias. It also lacks an exposure denominator for the drug-treated population (e.g., total number of people using the drug or number of exposed person-years). The frequency of reports is not equated with the true incidence or relative risk. Third, disproportionality analysis is purely for signal detection and is not causal. The time to market, market penetration, and clinical use of leflunomide, baricitinib, and upadacitinib differed significantly. AE reports were concentrated in North America and may not represent global usage. In addition, no confounding factors such as concomitant medications, comorbidities, disease activity, and treatment response were assessed. Some PT-level signals, for example, joint pain, fatigue, hospitalization, and interruption of treatment, can reflect the presence or failure of RA activity or treatment failure rather than drug toxicity. Furthermore, FAERS cannot distinguish between these sources. The numerous missing data for variables such as age and weight and can lead to selection bias in the subgroup analyses. The small number of reports regarding baricitinib limit the stability of some subgroup estimates (large CIs). Time trends were not supported by actual drug use or prescription data and hence remain speculations.

Future integration of multi-regional databases such as FAERS, EudraVigilance, and JADER together with links to electronic health records and insurance claims will provide the exposure denominators needed to calculate standard AE rates (e.g., annual reporting rates per 1,000 patients). Based on these data, a prospective cohort design may be developed that includes active comparators (e.g., users of standard synthetic antirheumatic drugs) and controls confounded by propensity score matching to validate signals identified in FAERS and estimate relative risks. Causality assessment may then be performed using established tools like the Naranjo algorithm, RUCAM or WHOUMC causality assessment systems. For missing data on variables such as age and weight, electronic health records or registration databases that harbor fewer missing data, and application of multiple imputations should be used to improve the robustness of subgroup estimates. For signals of high concern (infections in older patients, baricitinib-related infections, upadacitinib-related cardiovascular events, and COVID-19 signals), cell and animal experiments should be performed to better understand the responsible molecular mechanisms.

## 5 Conclusion

This study summarized the AE signals for leflunomide, baricitinib, and upadacitinib in patients with RA using the FAERS database. Leflunomide showed disproportionality signals for hepatobiliary disorders, baricitinib for infections and abnormalities, and upadacitinib for severe infections such as COVID-19 and myocardial infarction. Sex-based stratification revealed that female patients comprised more than 75% of all AE reports. Age stratification indicated that baricitinib reports were more frequent in older patients, whereas leflunomide and upadacitinib were reported to be more common among adults aged 18–64.9 years. In clinical practice, enhanced infection screening and routine hematological monitoring are recommended for older patients receiving baricitinib. For patients treated with leflunomide, liver function should be assessed and dose adjustments considered. When prescribing upadacitinib, clinicians should be vigilant for cardiovascular events and severe infections. To reduce AEs, we recommend pre-treatment assessment, regular laboratory surveillance, and patient education. Future studies should employ prospective designs and causal assessment methods to validate these findings.

## Data Availability

The original contributions presented in the study are included in the article/Supplementary material, further inquiries can be directed to the corresponding author.

## Footnotes

1
https://fis.fda.gov/extensions/FPD-QDE-FAERS/FPD-QDE-FAERS.html

2
https://CRAN.R-project.org/package=readxl

## Supplementary material

SUPPLEMENTARY TABLE S1 Computational formula.

SUPPLEMENTARY TABLE S2 The basic characteristics of ADE related to leflunomide, upadacitinib, and baricitinib in the treatment of RA in the FAERS database.

SUPPLEMENTARY TABLE S3 Strength of reported signals (SOC level) of baricitinib and RA in the FAERS database.

SUPPLEMENTARY TABLE S4 Strength of reported signals (SOC level) for leflunomide and RA in the FAERS database.

SUPPLEMENTARY TABLE S5 Strength of reported signals (SOC level) for upadacitinib in relation to RA in the FAERS database.

SUPPLEMENTARY TABLE S6 Strength of reported signals for baricitinib and RA in the FAERS database (PT level).

SUPPLEMENTARY TABLE S7 Strength of reported signals for leflunomide and RA in the FAERS database (PT level).

SUPPLEMENTARY TABLE S8 Strength of reported signals for upadacitinib and RA in the FAERS database (PT level).

SUPPLEMENTARY FIGURE S1 Temporal trend line chart of adverse drug events (ADEs) related to rheumatoid arthritis (RA) treatment with three drugs. Different colors represent different drugs, and larger dots indicate a higher number of ADE reports.
